# Supplementary material for: Salvage versus Primary Liver Transplantation for Hepatocellular Carcinoma: A Twenty-Year Experience Meta-Analysis
Source: Cancers (Basel). 2022 Jul 16;14(14):3465. doi: 10.3390/cancers14143465 (PMC9320001; doi:10.3390/cancers14143465)
Supplement: Supplementary file 1 [file cancers-14-03465-s001.zip › Supplementary File S3.pdf]

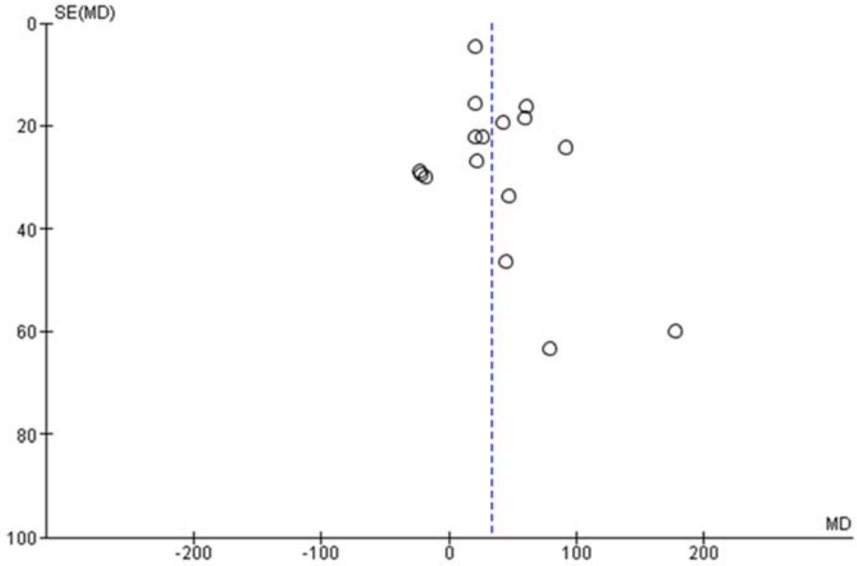

Figure S1. - Operating time.

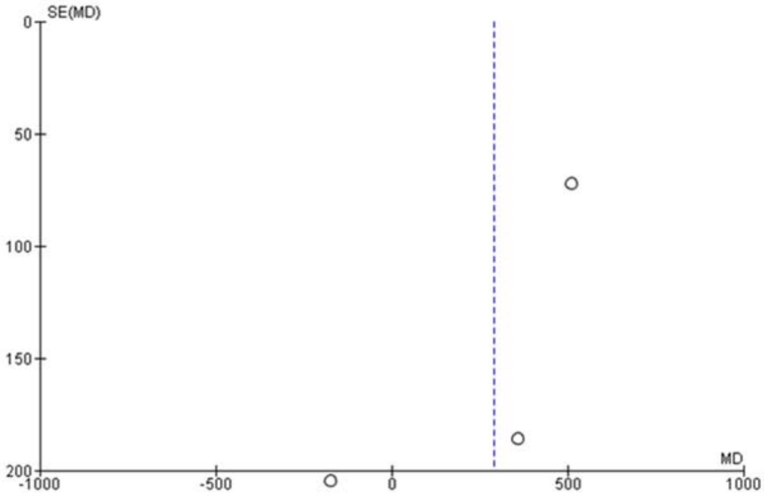

Figure S2. - Intraoperative blood loss .

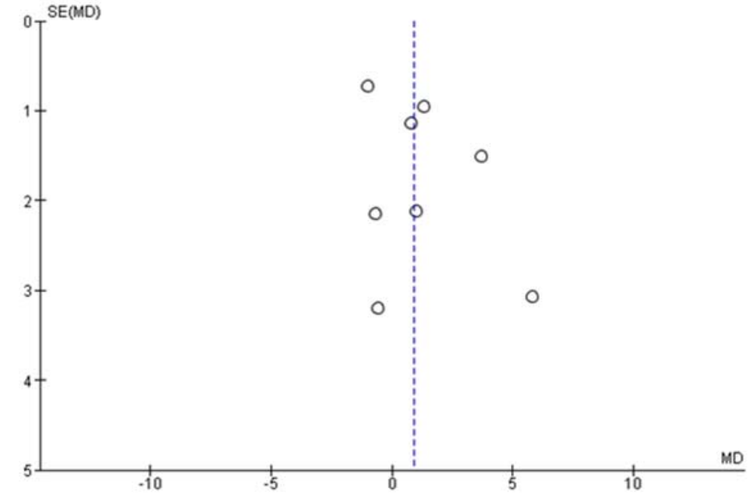

Figure S3. - Intraoperative Red Blood Cell (RBC) transfusion.

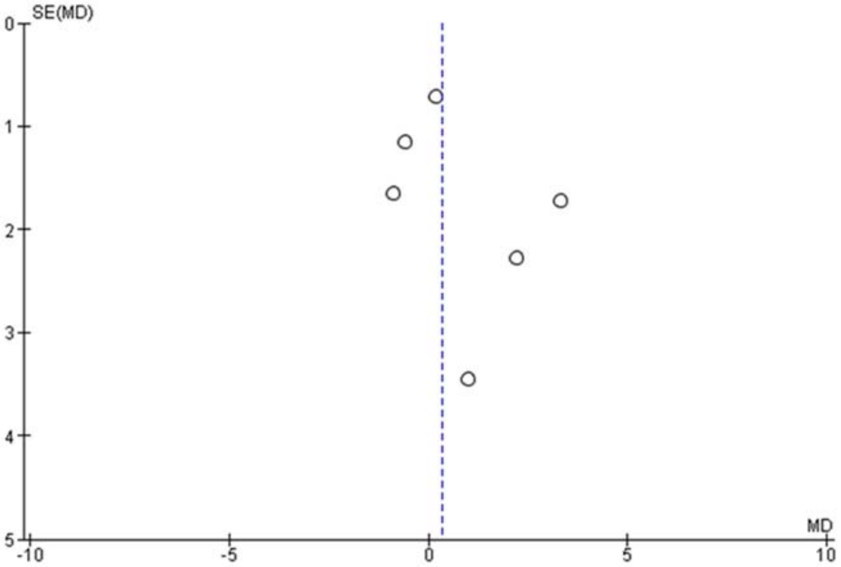

Figure S4 - Fresh frozen plasma (FFP) transfusion.

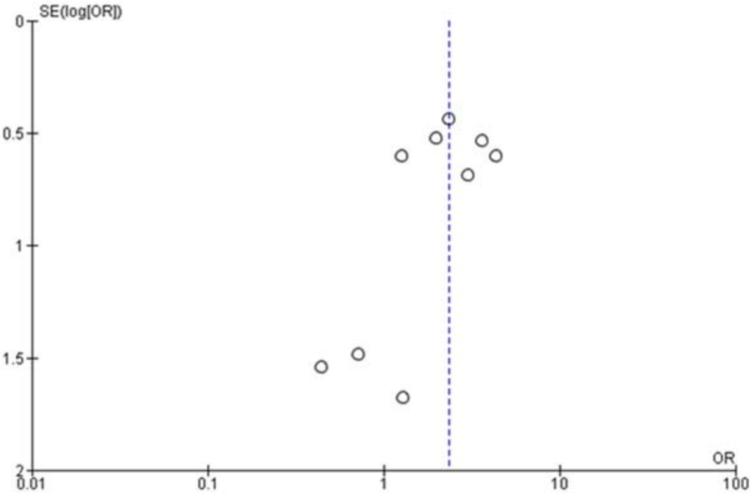

Figure S5. - Reoperation rate.

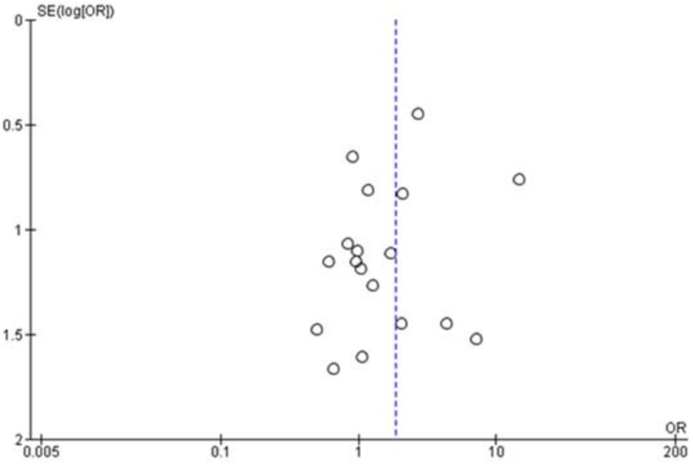

Figure S6. - Perioperative mortality rate .

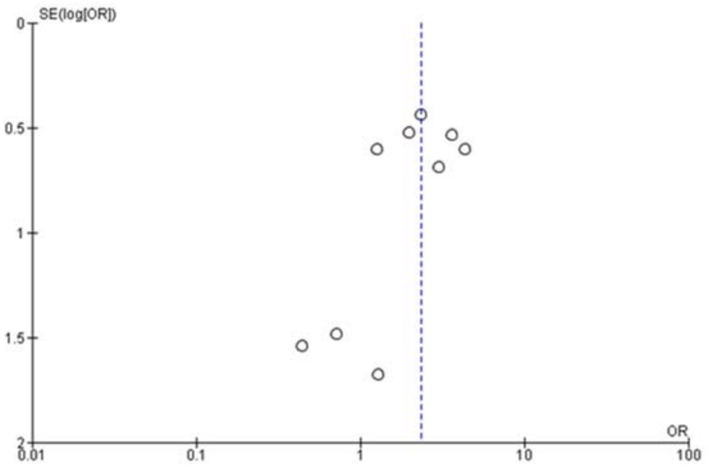

Figure S7. - Re-transplantation rate.

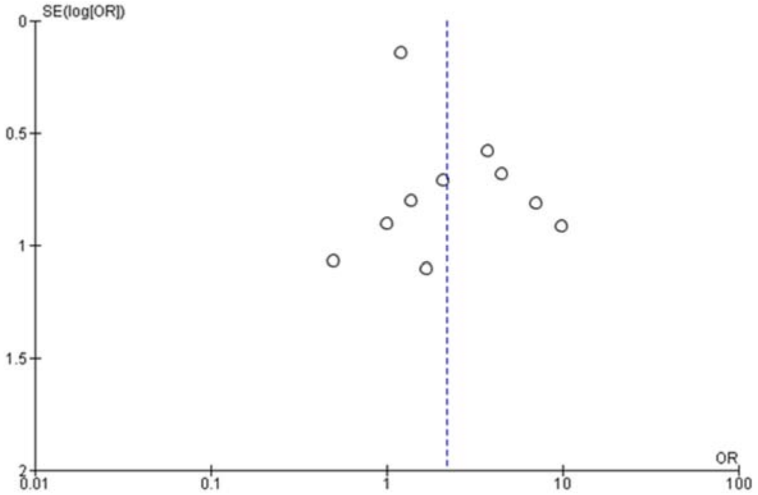

Figure S8. - Postoperative bleeding .

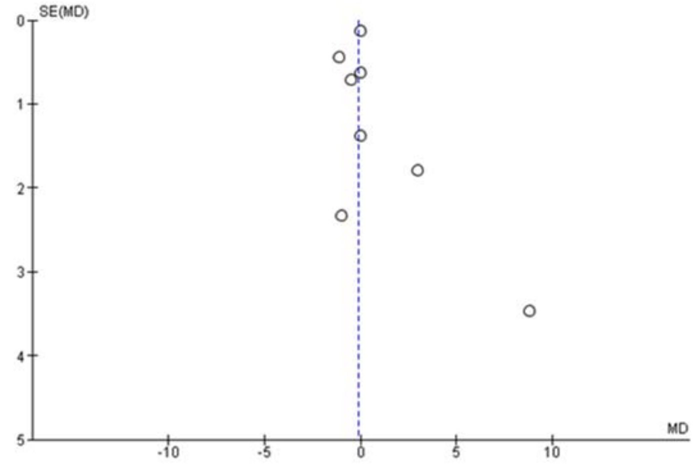

Figure S9. - Intensive Care Unit stay.

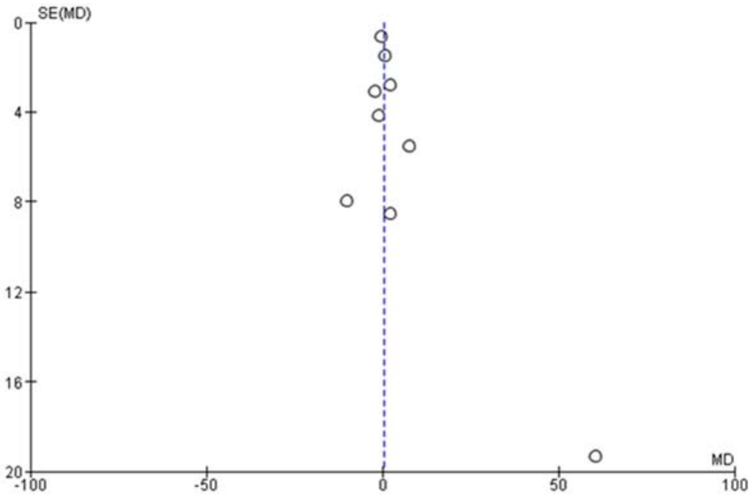

Figure S10. - Length of Hospital stay .

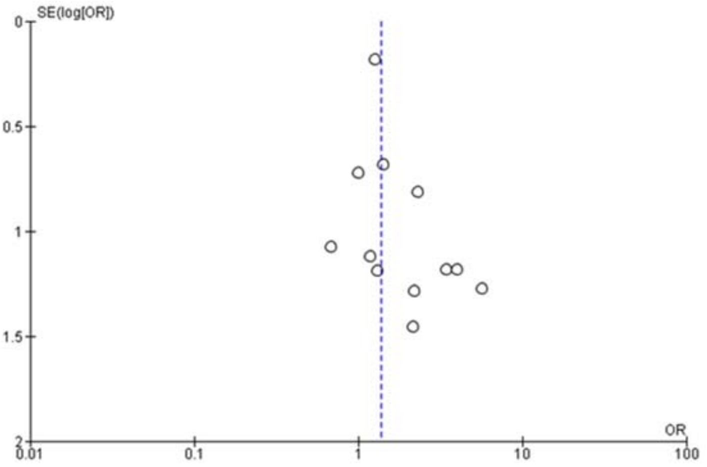

Figure S11. - Overall vascular complication.

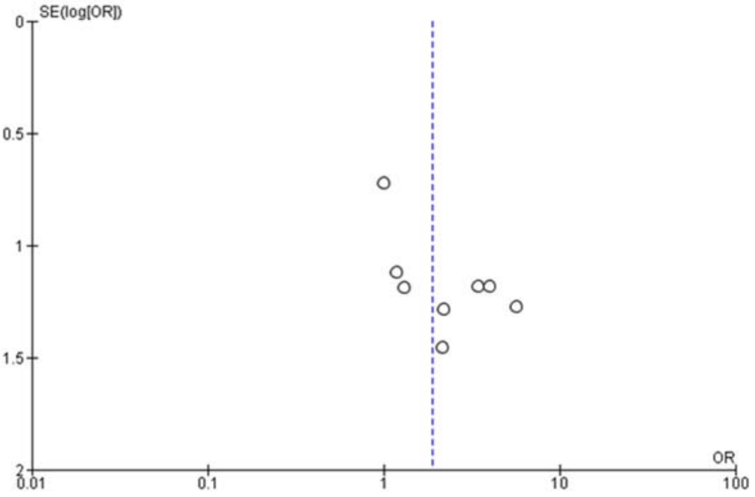

Figure S12. - Arterial thrombosis.

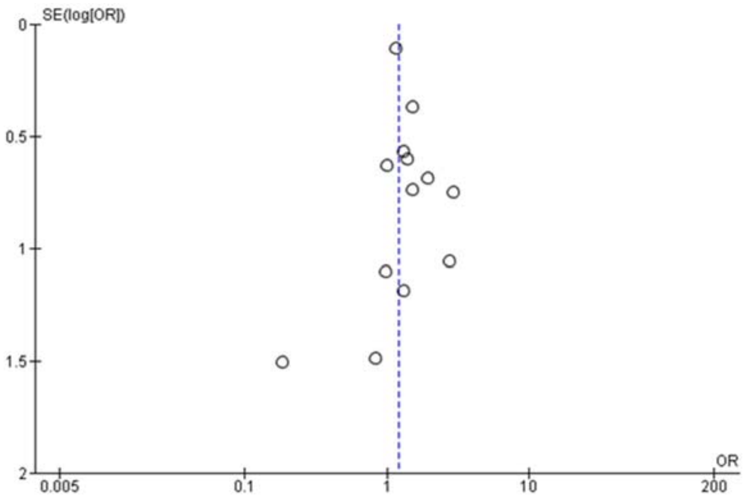

Figure S13. - Biliary complication.

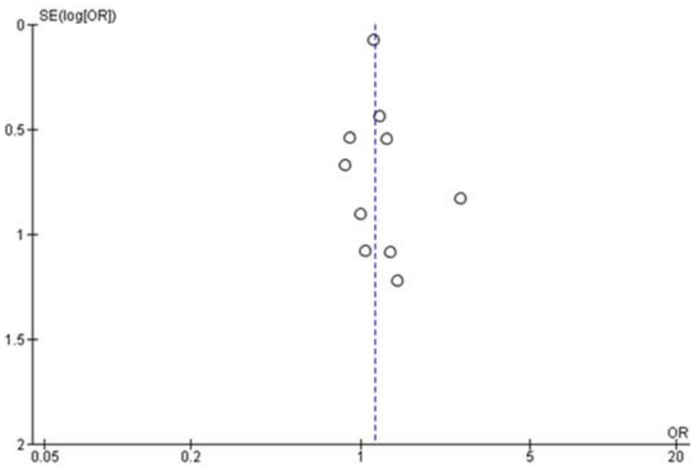

Figure S14. - Infection and sepsis .

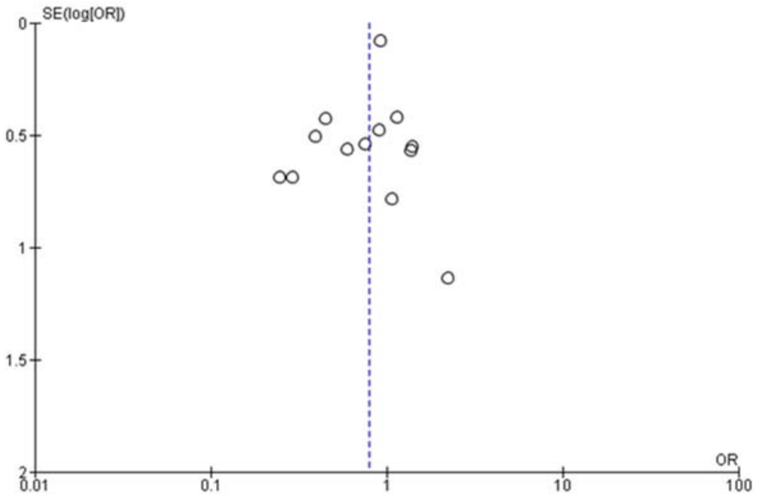

Figure S15. - 1y-Overall survival rates.

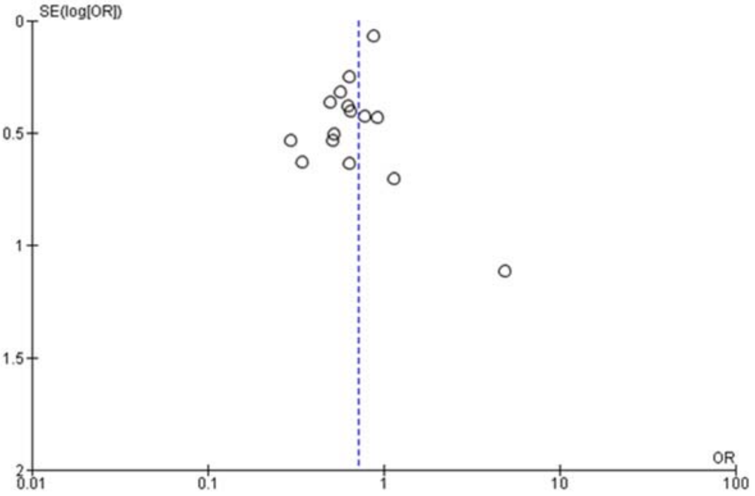

Figure S16. - 3y-Overall survival rates.

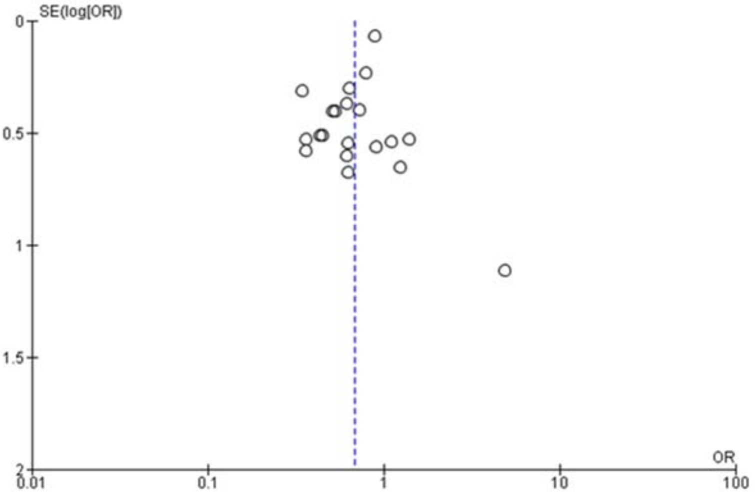

Figure S17. - 5y-Overall survival rates.

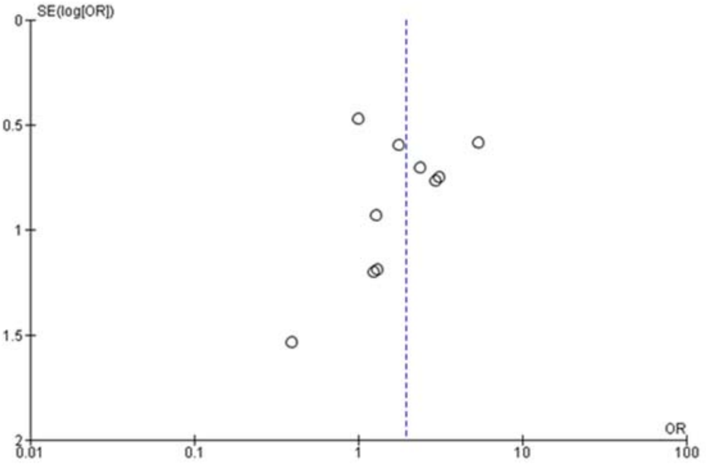

Figure S18. - HCC recurrence rate.

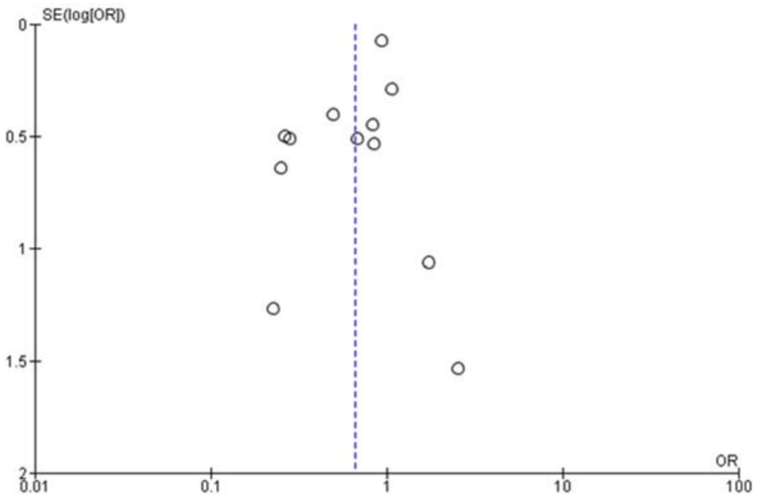

Figure S19. - 1y-Disease free survival rates.

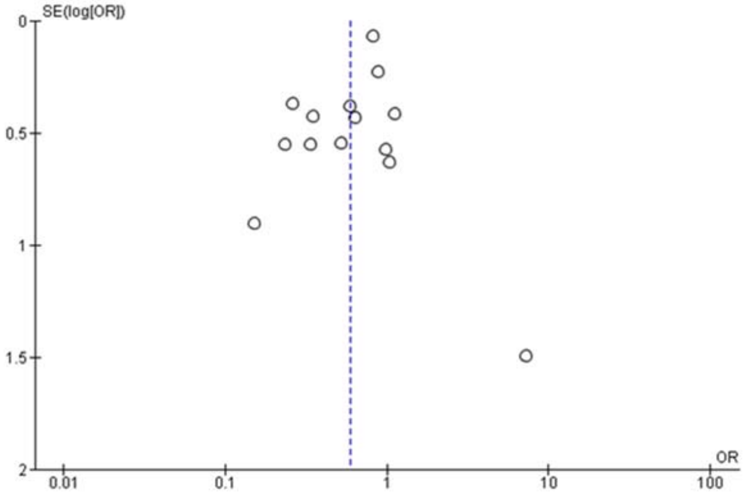

Figure S20. - 3y-Disease free survival rates.

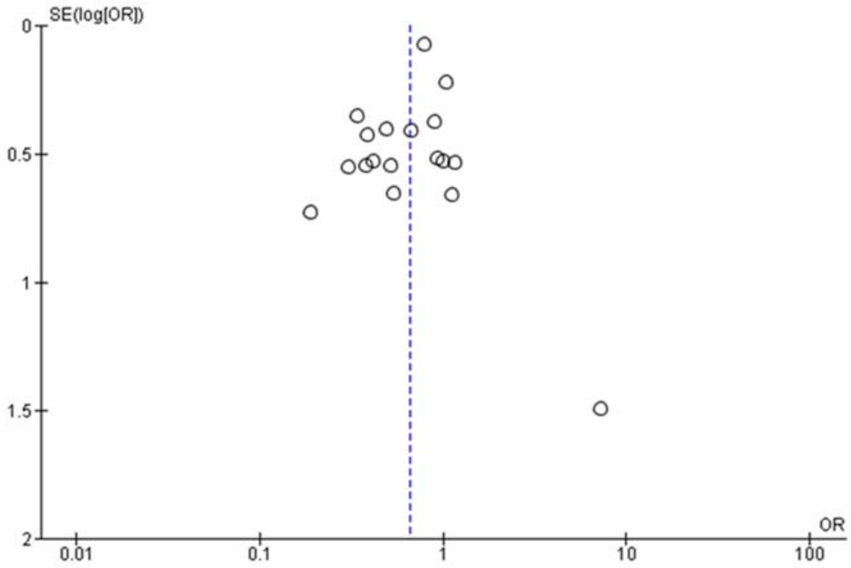

Figure S21. - 5y-Disease free survival rates.
